# Comorbidity in patients with first-ever ischemic stroke: Disease patterns and their associations with cognitive and physical function

**DOI:** 10.3389/fnagi.2022.887032

**Published:** 2022-09-09

**Authors:** Rui She, Zhongrui Yan, Yanlei Hao, Zuoji Zhang, Yifeng Du, Yajun Liang, Davide L. Vetrano, Joost Dekker, Bo Bai, Joseph T. F. Lau, Chengxuan Qiu

**Affiliations:** ^1^Department of Rehabilitation Sciences, The Hong Kong Polytechnic University, Hong Kong, Hong Kong SAR, China; ^2^JC School of Public Health and Primary Care, The Chinese University of Hong Kong, Hong Kong, Hong Kong SAR, China; ^3^Department of Neurology, Jining No. 1 People’s Hospital, Jining, Shandong, China; ^4^Department of Neurology, The Affiliated Hospital of Jining Medical University, Jining, Shandong, China; ^5^Department of Neurology, Jining Medical University, Jining, Shandong, China; ^6^Department of Neurology, Shandong Provincial Hospital Affiliated to Shandong University, Jinan, Shandong, China; ^7^Department of Global Public Health, Karolinska Institutet, Stockholm, Sweden; ^8^Aging Research Center, Department of Neurobiology, Care Sciences and Society, Karolinska Institutet and Stockholm University, Stockholm, Sweden; ^9^Centro Medicina dell’Invecchiamento, Fondazione Policlinico “A. Gemelli” IRCCS and Catholic University of Rome, Rome, Italy; ^10^Department of Psychiatry and Department of Rehabilitation Medicine, Amsterdam University Medical Centers, Amsterdam, Netherlands; ^11^Zhejiang Provincial Clinical Research Center for Mental Disorders, The Affiliated Wenzhou Kangning Hospital, Wenzhou Medical University, Wenzhou, China; ^12^School of Mental Health, Wenzhou Medical University, Wenzhou, China

**Keywords:** stroke, comorbidity, functional dependence, cognitive impairment, China

## Abstract

The present study examined the prevalence and pattern of comorbidity among Chinese patients with first-ever acute ischemic stroke, and assessed the associations of specific comorbidity patterns with physical and cognitive functioning after stroke occurrence. A hospital-based cross-sectional study was conducted among 2,151 patients with first-ever ischemic stroke (age ≥40 years; 64.2% men) who were admitted to two university hospitals in Shandong, China between 2016 and 2017. Data on demographics, lifestyles, chronic health conditions, and use of medications were collected through in-person interviews, clinical examinations, and laboratory tests. Physical functioning was assessed by the Barthel index (BI) and the modified Rankin Scale (mRS) while cognitive functioning was assessed by the Montreal Cognitive Assessment test. The results showed that comorbidity was present in 90.9% of the stroke patients (women vs. men: 95.2 vs. 88.7%, *P* < 0.001). Exploratory factor analysis identified three patterns of comorbidity, i.e., patterns of degenerative-cardiopulmonary, heart-gastrointestinal-psychiatric, and metabolic-kidney diseases. The number of comorbidities was significantly associated with a higher likelihood of moderate-to-severe physical dependence [odds ratio (95% CI) = 1.15 (1.06–1.25) for BI and 1.12 (1.04–1.21) for mRS, all *P* < 0.01] and cognitive impairment [odds ratio (95% CI) = 1.11 (1.02–1.20), *P* = 0.017], after adjusting for multiple covariates. Almost all the three comorbidity patterns were associated with increased likelihoods of physical dependence (range for odds ratios: 1.26–1.33) and cognitive impairment (range for odds ratios: 1.25–1.34). No significant association was found between degenerative-cardiopulmonary pattern and mRS. These findings suggest that comorbidity is associated with poor physical and cognitive functioning during the acute phase of ischemic stroke. Routine assessments of comorbidity and cognitive and physical function among patients with acute ischemic stroke should be considered in stroke research and clinical practice.

## Introduction

Stroke is the second leading cause of disability and death worldwide. In China, ∼2.4 million new stroke cases and ∼1.1 million stroke-related deaths occurred in 2013 (Wu et al., 2019), and the numbers are expected to rise over the next few decades primarily due to population growth and aging. Survivors of stroke often present with poor physical function and impaired cognition, leading to significantly decreased quality of life (Davis et al., 2019; She et al., 2021). Approximately 70–80% of stroke survivors require rehabilitation and long-term care (Nichols-Larsen et al., 2005). Post-stroke physical and cognitive conditions have been associated with demographics (e.g., age and gender), stroke features (e.g., size, location, and type), walking capacity, and psychosocial factors (e.g., social support, depression, and balance self-efficacy) (Salbach et al., 2006; Yoon et al., 2017; Ilunga Tshiswaka et al., 2018). As post-stroke function deficits may increase the risk of readmission, mortality, and early death (Gaynor et al., 2018), exploring factors associated with poor functional status among survivors of patients with stroke is greatly warranted if we seek to achieve the overall goal of rehabilitation services.

Stroke survivors commonly have comorbid conditions, especially older stroke patients. Evidence suggests that a clinical stroke could occur in the absence of comorbid conditions in less than 6% of cases (Nelson et al., 2017). Multimorbidity, the co-occurrence of two or more chronic diseases in the same person, is sometimes used interchangeably with comorbidity, which is defined as the presence of at least one long-term condition alongside an index condition (Gallacher et al., 2019). The coexisting of multiple chronic conditions and the presence of comorbidity patterns may be due to the fact that these health conditions share common risk factors and pathophysiology (e.g., heart disease and stroke) or causal/precursor relationship (e.g., atrial fibrillation and stroke) (Schäfer et al., 2010; Gallacher et al., 2019). Previously, community-based studies indicated that the burden and patterns of multimorbidity were associated with impaired physical functioning, poorer quality of life, and more frequent use of health care services (Ryan et al., 2015; She et al., 2019). However, there is a dearth of studies that have focused specifically on comorbidity patterns in the stroke population. We only identified a few studies that investigated the associations between comorbidity (mainly operationalized as a count of numbers of chronic health conditions or comorbidity index) and poor physical function among stroke patients, which reported mixed findings (Schmidt et al., 2014; Kabboord et al., 2016; Gallacher et al., 2019).

Thus, in this hospital-based cross-sectional study, we aimed to investigate the burden and pattern of chronic disease comorbidity among Chinese patients with first-ever ischemic stroke; and further to explore the associations between the number and patterns of comorbidity and physical and cognitive function outcomes among stroke patients.

## Materials and methods

### Patients

The participants were derived from a randomized controlled multimodal behavioral intervention trial among patients with acute ischemic stroke or transient ischemic attacks (TIAs), who were hospitalized in two university hospitals in Jining, Shandong, China. The inclusion criteria were (Wang et al., 2011): (1) first-ever ischemic stroke or TIAs that was confirmed by brain computed tomography (CT) or magnetic resonance imaging (MRI) scans; (2) aged ≥40 years; (3) patients, family members or caregivers can provide consent. Patients with severe symptoms (e.g., unconsciousness or aphasia) were excluded. From January 2016 to February 2017, 2,187 eligible stroke patients were recruited and completed the assessments within 2 weeks of admission to hospitals. We excluded 36 (1.7%) participants who were diagnosed with dementia, leaving 2,151 persons for the current analysis.

### Ethical statement

The study protocols were approved by the Ethics Committee at the relevant institution (No. 2015B006). Written informed consent was obtained from all participants, or in the case of cognitively impaired persons, from informants. Research had been conducted in accordance with the ethical principles expressed in the Declaration of Helsinki. The trial was registered in the Chinese Clinical Trial Registry (ID: ChiCTR-IOR-16007741).

### Data collection

Baseline data were collected within 2 weeks after hospitalization when patients’ clinical conditions became stable, following the standardized questionnaire through face-to-face interviews, clinical examinations, psychological testing, and laboratory tests by trained staff at the two hospitals. We collected data on sociodemographics (e.g., age, sex, and education), lifestyles prior to hospitalization (i.e., smoking and alcohol drinking), use of medications in the 2 weeks before hospitalization, and cognitive and physical function following the hospitalization. Weight and height were measured in light clothes with no shoes. Body mass index was calculated as weight (kg) divided by height (meters) squared. Arterial blood pressure was measured in the sitting position on the right arm after at least a 5-min rest, using an electronic blood pressure monitor (Omron HEM-7127J, Omron Electronics Inc., Japan). Blood pressure was measured three times on one occasion, and the mean of the three readings was used in the analysis. Peripheral blood samples were obtained after an overnight fast. Fasting plasma glucose (FPG) and total cholesterol (TC) were measured using an automatic Biochemical Analyzer at the hospital laboratories.

### Assessments of health conditions

#### Chronic diseases and comorbidity

We defined and categorized 16 chronic disease categories following the methods previously described (Calderón-Larrañaga et al., 2017). We defined hypertension as blood pressure ≥140/90 mmHg or use of antihypertensive drugs (Song et al., 2014; Wang et al., 2015), diabetes as FPG ≥7.0 mmol/l or use of oral antidiabetic agents or insulin injection (American Diabetes Association, 2014), obesity as a body mass index ≥28 kg/m^2^ (Song et al., 2014), and dyslipidemia as total serum cholesterol >6.22 mmol/l or triglycerides ≥2.26 mmol/l or low density lipoprotein cholesterol ≥4.14 mmol/l or use of hypolipidemic drugs (Calderón-Larrañaga et al., 2017). Gastrointestinal diseases were ascertained as clinical diagnosis of gastric or duodenal ulcer or chronic gastritis or use of antacids. Respiratory diseases were ascertained by clinical diagnosis of chronic obstructive pulmonary disease (COPD) or asthma or use of antiasthmatic drugs. Kidney diseases were ascertained by clinical diagnosis of nephritis or kidney failure. Depression was defined as having a clinical diagnosis of major depression according to the structured clinical interview of the fifth edition of Diagnostic and Statistical Manual of Mental Disorders, significant depressive symptoms defined as the 15-item Geriatric Depression Scale (GDS-15) score ≥5 (Chan, 1996; Almeida and Almeida, 1999), or using antidepressants during the hospital admission. Cancer, coronary heart disease, migraine, fracture, heart failure, arrhythmia, cerebrovascular malformation, and arthritis were ascertained by integrating information from clinical examination, instrumental examination (e.g., electrocardiogram and B-mode ultrasonic examination), blood test, and discharge diagnosis. Chronic diseases with a prevalence of <0.5% were not included in the analysis in order to avoid spurious associations and obtain epidemiologically coherent patterns, such as brain injury (0.1%), epilepsy (0.2%), Parkinson’s disease (0.2%), and thyroid dysfunction (0.4%). Multimorbidity was defined as the co-occurrence of two or more diseases in the same individual; therefore, the presence of one or more of the 16 comorbidities in patients with stroke indicated the presence of multimorbidity.

#### Physical function

Physical function was evaluated using the Barthel Index (BI) (McLennan et al., 2011) and the modified Rankin Scale (mRS) (Banks and Marotta, 2007). The BI included 10 basic self-care activities, i.e., bowel control, bladder control, personal hygiene, toilet transfer, bathtub transfer, feeding, dressing, wheelchair transfer to and from bed, walking, and ascending and descending stairs. The total score ranged from 0 to 100, with higher scores indicating higher degrees of independence. The cutoff score of ≤75 was used to denote the presence of moderate-to-severe physical dependence in stroke patients (Supervía et al., 2008). The mRS is a commonly used scale for measuring the degree of disability or dependence in the daily activities of people who have suffered from a stroke or other causes of neurological disability. The mRS comprises 6 grades of stroke severity ranging from 0 (no significant disability) to 5 (severe disability). Unfavorable outcome on the mRS was defined as the score ≥3 (moderate-to-severe disability) (Diener et al., 2008).

#### Cognitive function

Global cognitive function was assessed with the validated Changsha version of Montreal Cognitive Assessment (MoCA) (Nasreddine et al., 2005). MoCA is a 30-point test, which measures language, memory, attention, abstraction, orientation, and executive functions. Based on a national population-based study among Chinese elderly people, cognitive impairment in patients with stroke was defined as MoCA score <14 for illiterate individuals, <20 for individuals with 1 to 6 years of education, and <25 for individuals with 7 or more years of education (Lu et al., 2011).

#### Covariates

Demographic features including age (years), sex, education (no formal schooling, primary school, and middle school or above), marital status (married vs. single or widowed or divorced), enrolled hospital, type of stroke (cerebral infarction, TIA, and lacunar infarction), and lifestyle factors (i.e., smoking, alcohol drinking, and whether or not having difficulty in falling asleep) were controlled as covariates.

### Statistical analysis

Characteristics of study participants by sex were present and compared using *t*-test for continuous variables or chi-square test for categorical variables. Exploratory factor analysis was performed to identify comorbidity patterns based on a tetrachoric correlation matrix and using principal factor analysis (Mislevy, 1985). Eigenvalues greater than 1 and the scree plot were used to determine the number of retained factors (Prados-Torres et al., 2012). A chronic condition was considered to characterize a given pattern of comorbidity if its loading was ≥0.25 in that pattern. When the factor loading of a certain disease was ≥0.25 in more than one group, this disease was clustered into the group with a larger factor loading value (Wang et al., 2015). To facilitate the interpretation of the factors, an oblique rotation (Oblimin) was applied. For each comorbidity pattern, we used regression method (a least squares regression approach) to estimate the factor scores of participants with respect to their factor loading values and the factor scores for each comorbidity pattern were divided into tertiles (Distefano et al., 2008). From the lowest to the highest tertile, the participant’s expression of the comorbidity pattern associated with the specific component increased.

Multiple logistic regression models were utilized to examine the associations between the number of comorbidities with physical and cognitive functional outcomes, respectively, controlling for sociodemographic variables, lifestyle factors, and type of stroke. A total of 526 patients (24.5%) had missing data on at least one item of studied variables, such as sociodemographic and lifestyle factors (16.0%), measurement of comorbidities (9.5%), the MoCA test (6.3%), BI (1.3%), and mRS (1.2%). Missing values were dealt with multiple imputations (*n* = 20), which shows advantages (e.g., reducing bias, increasing validity, and preserving statistical power) over most of the existing methods dealing with missing data (McCleary, 2002). Relative variance increased (RVI) as a measurement for evaluation of multiple imputation was reported, which is interpreted as the proportional increase in the sampling variance of the parameter of interest that is due to the missing data. The closer this number is to zero, the less effect missing data have on the variance of the estimate. The odds ratios (OR) and 95% confidence intervals (CIs) of cognitive or physical impairment associated with tertile of factor scores for each comorbidity pattern were also estimated, in which the first tertile was used as the reference category. SPSS 23.0 Statistics for Windows (IBM Corp., Released 2015, Armonk, NY, United States: IBM Corp.) and Stata Statistical Software: Release 12.0 (StataCorp 2011, College Station, TX, United States: StataCorp LP) were used for all statistical analyses. Two-tailed *P*-value < 0.05 was considered statistically significant.

## Results

### Characteristics of the study participants

**FIGURE 1:**
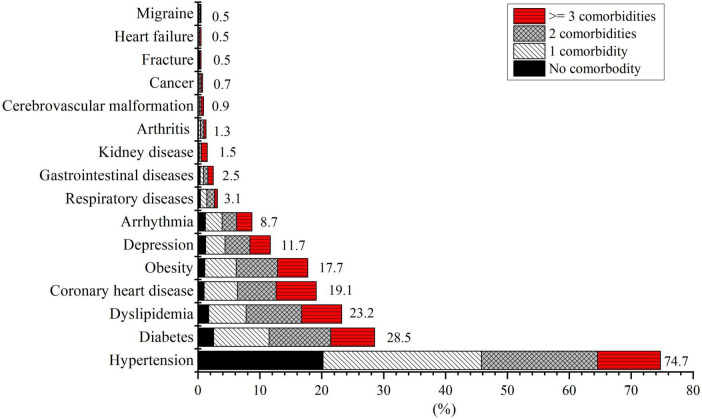
Prevalence (per 100 patient population) and co-occurrence of chronic diseases among patients with first-ever acute ischemic stroke (*n* = 2,151). Comorbidity refers to chronic diseases other than the specific condition and stroke.

The mean age of the 2,151 participants was 61.5 (SD, 9.8) years and 64.2% were men. Of all the stroke patients, 93.7% were diagnosed with cerebral infarction, 3.6% with TIAs, and 2.7% with lacunar infarction; 40.3% were current smokers; 36.3% had alcohol drinking habits in the past year, and 13.9% had difficulties in falling asleep. The average number of comorbidities per patient was 2.0 (SD 1.2). Only 9.0% did not present any other comorbidities, and 29.6, 30.1, and 31.3% had one, two, and three or more comorbidities, respectively. Multimorbidity affected 91.0% of all stroke patients. Compared with patients who had <2 comorbidities, stroke patients with two or more comorbidities were more likely to be female (72.1 vs. 55.4%, *P* < 0.001), have employee health insurance (24.7 vs. 18.6%, *P* < 0.001), have sleep problems (16.4 vs. 12.2%, *P* = 0.011) while less likely to be current smokers (35.6 vs. 48.5%, *P* < 0.001) and drink alcohol (33.3 vs. 42.8%, *P* < 0.001). No differences in age, marital status, and stroke subtypes were observed between patients with ≥2 and <2 comorbidities. Overall, the most common comorbidities in the stroke patients were hypertension (74.7%), diabetes (28.5%), dyslipidemia (23.3%), coronary heart disease (19.1%), and obesity (17.7%) (Figure 1).

Moderate-to-severe physical dependence defined by BI ≤75 or mRS ≥3 was found in 35.4 and 36.4% of the patients, respectively. Cognitive impairment was present in 57.5% of the patients. Female stroke patients were older, less educated, more likely to be widowed/divorced and have difficulties in falling asleep, less likely to smoke and drink alcohol, had more comorbidities, and were more likely to have multimorbidity and physical dependence (Table 1).

**TABLE 1:** Characteristics of the study participants.

Characteristics	Total sample (*n* = 2,151)	Men (*n* = 1,380)	Women (*n* = 771)	*P*-value*
**Sociodemographic and lifestyle characteristics**				
Age (years), mean (SD)	61.5 (9.8)	60.4 (9.7)	63.3 (9.8)	<0.001
Education^b^				
No formal school	545 (26.4)	137 (10.4)	408 (54.7)	<0.001
Primary school	544 (26.3)	366 (27.7)	178 (23.9)	
Middle school or above	979 (47.3)	819 (62.0)	160 (21.5)	
Marital status^b^				
Married	2,068 (96.7)	1,340 (97.8)	728 (94.7)	<0.001
Widowed/Divorced/Single	71 (3.3)	30 (2.2)	41 (5.3)	
Health insurance^a,^ ^b^				
Urban residents basic medical insurance	1,172 (55.7)	688 (50.8)	484 (64.5)	<0.001
Employee health insurance	466 (22.1)	372 (27.5)	94 (12.5)	
NRCMS health insurance	390 (18.5)	245 (18.1)	145 (19.3)	
Others (e.g., Public expense)	77 (3.7)	50 (3.7)	27 (3.6)	
Current smoking^b^				
No	1,269 (59.7)	550 (40.5)	719 (94.0)	<0.001
Yes	855 (40.3)	809 (59.5)	46 (6.0)	
Alcohol drinking^b^				
No	1,228 (63.7)	489 (41.8)	739 (97.2)	<0.001
Yes	701 (36.3)	680 (58.2)	21 (2.8)	
Difficulties in falling asleep^b^				
No	1,819 (86.1)	1,213 (89.5)	606 (80.1)	<0.001
Yes	293 (13.9)	142 (10.5)	151 (19.9)	
**Clinical characteristics**				
Stroke type				
Cerebral infarction	2,015 (93.7)	1,301 (94.3)	714 (92.6)	0.302
TIAs	77 (3.6)	44 (3.2)	33 (4.3)	
Lacunar infarction	59 (2.7)	35 (2.5)	24 (3.1)	
**Comorbidities** ^b^				
No. of chronic conditions, mean (SD)	2.0 (1.2)	1.8 (1.1)	2.3 (1.2)	<0.001
Multimorbidity (≥1 comorbidity)				
No	175 (9.0)	142 (11.3)	33 (4.8)	<0.001
Yes	1,771 (91.0)	1,112 (88.7)	659 (95.2)	
No. of comorbidities				
≤1	752 (38.6)	559 (44.6)	193 (27.9)	<0.001
2	585 (30.1)	376 (30.0)	209 (30.2)	
≥3	609 (31.3)	319 (25.4)	290 (41.9)	
**Physical and cognitive function**				
Physical dependence (Barthel Index ≤75)^b^				
No	1,373 (64.1)	907 (66.6)	466 (61.0)	0.009
Yes	752 (35.4)	454 (33.4)	298 (39.0)	
Physical disability (mRS ≥3)^b^				
No	1,350 (63.6)	897 (66.0)	453 (59.4)	0.002
Yes	773 (36.4)	463 (34.0)	310 (40.6)	
Cognitive impairment (MoCA)^b^				
No	857 (42.5)	553 (42.7)	304 (42.2)	0.803
Yes	1,158 (57.5)	741 (57.3)	417 (57.8)	

Data are n (%), unless otherwise specified. MoCA, Montreal Cognitive Assessment; mRS, modified Rankin Scale. ^a^Urban resident basic medical insurance was a government-subsidized, household-level voluntary medical insurance for urban residents that were not covered by employee health insurance. Employee health insurance was for urban working and retired employees in public and private sectors, in which employers and employees contributed to the insurance system in proportion to the employee’s salary. New Rural Co-operative Medical Scheme (NRCMS) was for rural residents provided by local and central governments covering various rates of expense in all level public healthcare facilities. ^b^The number of patients with missing values was 83 for education, 12 for marital status, 46 for health insurance, 27 for current smoke, 222 for alcohol drinking, 39 for difficulties in falling asleep, 205 in comorbidities, 26 for BI, 29 for mRS, and 136 for MoCA. Missing values were dealt with multiple imputations (n = 20) in the regression analysis. *P value is for the test of difference between men and women.

### Patterns of chronic comorbidity

We identified three patterns of chronic comorbidity among stroke patients, which could explain 63.7% of the total variance (Table 2). Pattern 1 was referred to as degenerative-cardiopulmonary disease pattern, which included degenerative disorders (arthritis, fracture, and cancer), heart failure, respiratory diseases, and other diseases (migraine and cerebrovascular malformation). Pattern 2 was called as heart-gastrointestinal-psychiatric disease pattern, which included heart diseases (coronary heart disease and arrhythmia), gastrointestinal diseases, and depression. Pattern 3 was referred to as metabolic-kidney disease pattern that included metabolic disorders (hypertension, obesity, diabetes, and dyslipidemia) and kidney disease.

**TABLE 2:** Rotated loadings for each of the 16 chronic disease categories by three groups from factor analysis.

Chronic diseases	Degenerative-cardiopulmonary diseases	Heart- gastrointestinal-psychiatric diseases	Metabolic-kidney diseases
Fracture	**0.84**	−0.10	0.20
Arthritis	**0.58**	−0.15	−0.06
Heart failure	**0.57**	0.40	−0.32
Respiratory diseases	**0.46**	−0.09	−0.46
Migraine	**0.38**	0.35	−0.11
Cancer	**0.35**	0.23	−0.12
Cerebrovascular malformation	**0.29**	0.25	0.07
Coronary heart disease	−0.15	**0.76**	0.07
Gastrointestinal diseases	0.12	**0.35**	−0.02
Arrhythmia	−0.09	**0.32**	−0.24
Depression	0.04	**0.29**	0.06
Diabetes	0.15	−0.03	**0.73**
Dyslipidemia	−0.02	0.20	**0.43**
Kidney disease	0.33	0.34	**0.40**
Obesity	−0.07	−0.13	**0.26**
Hypertension	−0.22	0.22	**0.26**
**Eigenvalue**	2.65	1.42	1.20
**Cumulative percentage**	32.1%	49.2%	63.7%

Values in bold indicate the factor loadings of diseases that were assigned to the corresponding comorbidity pattern.

### Comorbidity, physical function, and cognitive function

The increasing number of comorbidities was associated with an increased likelihood of physical dependence [OR = 1.15 for BI and 1.12 for mRS; *P* < 0.01 for all] and cognitive impairment (OR = 1.11; *P* = 0.017) (Table 3). The presence of multimorbidity was positively associated with physical dependence assessed by BI (OR = 1.56; *P* = 0.013) and mRS (OR = 1.41; *P* = 0.049). Consistently, participants with two comorbidities and three or more comorbidities (OR = 1.34 and 1.56 for BI and OR = 1.28 and 1.51 for mRS, respectively; *P* < 0.05 for all) were more likely to have physical dependence than those who had no or only one comorbidity. Similarly, compared to participants who had no or only one comorbidity, those with three or more comorbidities were more likely to have cognitive impairment (OR = 1.36; *P* = 0.011) (Table 3).

**TABLE 3:** Associations of physical and cognitive functions with number of chronic health conditions.

Chronic health conditions	Moderate-to-severe physical dependence				Cognitive impairment	
		
	Barthel Index ≤75		mRS ≥3		Low MoCA score^a^	
			
	OR (95% CI)	*P*-value	OR (95% CI)	*P*-value	OR (95% CI)	*P*-value
No. of comorbidities	**1.15 (1.06, 1.25)**	**0.001**	**1.12 (1.04, 1.21)**	**0.005**	**1.11 (1.02, 1.20)**	**0.017**
Multimorbidity						
No	1.00 (reference)		1.00 (reference)		1.00 (reference)	
Yes	**1.56 (1.10, 2.23)**	**0.013**	**1.41 (1.00, 1.99)**	**0.049**	1.19 (0.84, 1.69)	0.336
No. of comorbidities						
≤1	1.00 (reference)		1.00 (reference)		1.00 (reference)	
2	**1.34 (1.05, 1.71)**	**0.019**	**1.28 (1.01, 1.61)**	**0.042**	1.15 (0.91, 1.45)	0.240
≥3	**1.56 (1.23, 1.98)**	**<0.001**	**1.51 (1.19, 1.94)**	**0.001**	**1.36 (1.07, 1.72)**	**0.011**

Odds ratio (95% confidence interval) was adjusted for age, sex, education level, marital status, type of health insurance, enrolled hospital, type of stroke, smoking status, alcohol drinking, and difficulties in falling asleep. Significant odds ratios (95% confidence interval) with P < 0.05 are presented in bold. OR, odds ratio; CI, confidence interval; mRS, modified Rankin Scale; McCA, Montreal Cognitive Assessment (Changsha version). ^a^Cognitive impairment in patients with stroke was defined as MoCA score <14 for illiterate individuals, <20 for individuals with 1 to 6 years of education, and <25 for individuals with 7 or more years of education.

**FIGURE 2:**
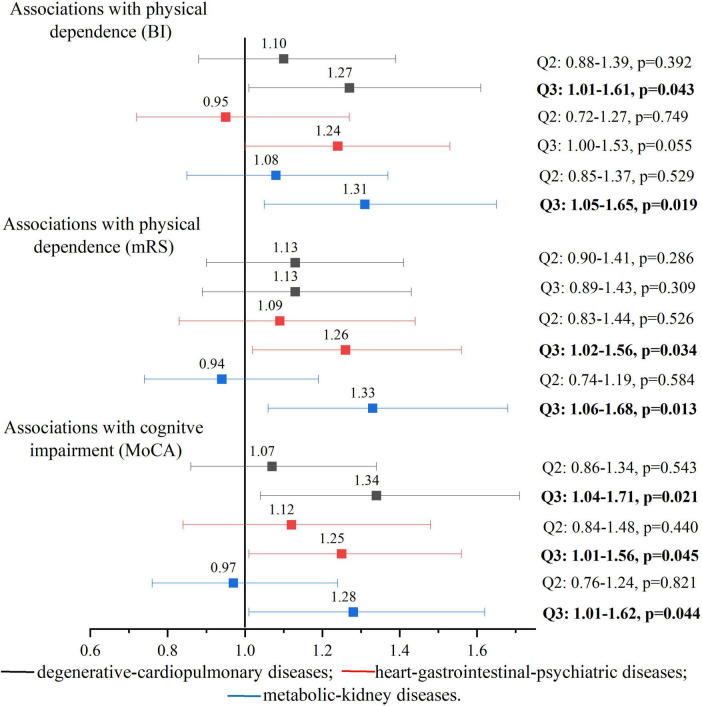
Associations between comorbidity patterns and functional outcomes among patients with first-ever ischemic stroke. Odds ratios and 95% confidence intervals were adjusted for age, sex, education, marital status, type of health insurance, enrolled hospital, type of stroke, smoking status, alcohol drinking, and difficulties in falling asleep. The lower tertile (Q1) was used as the reference group; Q2: the medium tertile; Q3: the upper tertile. Missing data were dealt with multiple imputation and the RVI for multiple logistic regression models ranged from 0.0298 to 0.0388.

Of the three comorbidity patterns, the upper tertile of degenerative-cardiopulmonary disease pattern (vs. lower tertile) was significantly associated with physical dependence assessed by BI [OR (95% CI) = 1.27 (1.01–1.61)] and cognitive impairment [OR (95% CI) = 1.34 (1.04–1.71)]. Compared to participants in the lower tertile of heart-gastrointestinal-psychiatric disease pattern, those in the upper tertile had a higher likelihood of physical dependence [OR (95% CI) = 1.24 (1.00–1.53) for BI and 1.26 (1.02–1.56) for mRS] and cognitive impairment [OR (95% CI) = 1.25 (1.01–1.56)]. Similarly, participants in the upper tertile of metabolic-kidney disease pattern were more likely to have physical dependence [OR (95% CI) = 1.31 (1.05–1.65) for BI and 1.33 (1.06–1.68) for mRS] and cognitive impairment [OR (95% CI) = 1.28 (1.01–1.62)] than those in the lower tertile. There was no significant association between the comorbidity pattern of degenerative-cardiopulmonary diseases and functional outcome assessed with mRS (Figure 2).

## Discussion

In this hospital-based study, comorbidity was present in nine out of the ten patients with first-ever acute stroke. The greater number of comorbidities was associated with an increased likelihood of physical dependence and cognitive impairment. Furthermore, three patterns of comorbidity were identified among stroke patients, which generally showed significant positive associations with physical dependence and cognitive impairment. To the best of our knowledge, this is the first study in a large sample of Chinese stroke patients that investigates the burden and patterns of comorbidity as well as their associations with cognitive and physical functional outcomes, which provides a novel perspective to assess the health impacts of comorbidities on post-stroke functions.

The overall prevalence of comorbidity was generally comparable with that of the previous examination in stroke patients, which showed great variations across studies due to differences in methodology (e.g., sample characteristics and the number, definition, and measurement of morbidities) (Gallacher et al., 2014). Patients with two or more comorbidities were more likely to have sleep problems while less likely to be current smokers and drink alcohol in the past year than those having no or only one comorbidity. Stroke patients with more comorbidities may be more inclined to be recommended by their doctors to adopt a healthy lifestyle prior to the occurrence of clinical stroke. The vulnerability of sleep disorders in stroke patients with multiple comorbidities warrants attention and further investigation. Corroborating previous studies, female patients were more likely to have poor physical functional status following an acute stroke (Gargano and Reeves, 2007). In the present study, female patients were older, less educated, and had more comorbidities and higher prevalence of multimorbidity, which might contribute to worse physical functioning. This suggests that greater attention should be paid to female stroke survivors to properly manage comorbidities and physical dependence.

We further identified three patterns of comorbidity among the stroke patients (i.e., degenerative-cardiopulmonary, heart-gastrointestinal-psychiatric, and metabolic-kidney diseases) and assessed their relationships with functional outcomes. The first pattern was mainly characterized by degenerative diseases (e.g., joint diseases), heart failure, cancer, and respiratory diseases. Relatedly, a previous study of patients in the primary care setting in Spain identified a comorbidity pattern of psychogeriatric diseases, which covered geriatric diseases, heart failure, stroke, and neurocognitive diseases (Prados-Torres et al., 2012). Another observational study based on electronic health records also suggested that the pattern of degenerative diseases (e.g., arthropathy, cataract, osteoporosis, and hearing loss) was one of the most prevalent comorbidity patterns in Spanish patients with heart failure, possibly explained by the shared risk of aging and physical limitation (Gimeno-Miguel et al., 2019). Corroborating a prior study among the Chinese elderly population, cancer was clustered with degenerative diseases, reflecting the strong age dependence of these conditions (Gu et al., 2017). A community-based survey of older adults in China also identified the comorbidity pattern of cancer and pulmonary diseases and suggested that cancer tended to affect most frequently the respiratory tracts (Wang et al., 2017). While no prior studies have explored comorbidity patterns among stroke patients, future studies are warranted to confirm whether this comorbidity pattern remains across different populations and geographic regions.

The second pattern was represented by cardiovascular diseases, gastrointestinal diseases, and depression. The close associations between gastrointestinal diseases and affective disorders have been frequently reported in the general and clinical populations (Mayer et al., 2001). Acute life-threatening stressors and the central nervous system mechanisms play an important role in the development of gastrointestinal and psychiatric symptoms. It is worth noting that cardiovascular and gastrointestinal diseases as a separate pattern was not common in previous studies. However, the recently discovered contribution of gut-microbiota-derived molecules in the development of heart disease and its risk factors has significantly increased attention toward the connection between gut and heart diseases (Ahmadmehrabi and Tang, 2017). For instance, gastroesophageal reflux disease can lead to atrial fibrillation, in which multiple mechanisms may be involved in such as inflammation, autoimmunity, and exacerbated autonomic stimulation (Martins et al., 2015). Future research should focus on the underlying pathogenesis connecting these medical conditions, and the role of age and aging in the development of cardiovascular and gastrointestinal diseases.

Consistent with prior literature of studies among the community-dwelling elderly people (Wang et al., 2015), hypertension, diabetes, dyslipidemia, and obesity, the main components of metabolic syndrome, are established risk factors for cardiovascular diseases. This finding corroborates a prior systematic review that identified the major multimorbidity pattern of cardiovascular-metabolic diseases in the adult populations (Prados-Torres et al., 2014). Additionally, we found that kidney disease was clustered with metabolic factors and disorders. Extensive literature has indicated that metabolic syndrome and its individual components (e.g., obesity and diabetes) are associated with heightened risks of chronic kidney diseases (Kumar et al., 2013). For instance, it is known that chronic high blood pressure is a major cause of kidney disease, while chronic kidney disease is one of the most common causes of secondary hypertension (Freedman and Cohen, 2016). Patients with long-term hypertension (e.g., >5 years) were more likely to suffer from kidney disease with the small renal arteries of the glomerulus pathological changes (Mulè et al., 2008). The epidemiologic data also revealed that the prevalence of diabetic kidney disease increased with the growing epidemic of diabetes (Harjutsalo and Groop, 2014). Therefore, kidney disease should be recognized and prevented as an important complication of cardiovascular and metabolic diseases. Our study indicated that these metabolic diseases were among the most prevalent comorbidities among stroke patients. Metabolic syndromes are arguably prominent risk factors in the development of stroke and thus they are common comorbid diseases among patients with stroke.

Furthermore, we found that the number of comorbidities was associated with an increased likelihood of both physical dependence and cognitive impairment among stroke survivors, which is consistent with literature among the general older population (She et al., 2019). Notably, the associations between comorbidity and poor physical function were overall consistent when using the two different tools for assessment of post-stroke functional outcomes (i.e., BI and mRS), supporting the robustness of the findings. The results corroborate a previous study showing that comorbidity assessed using the Charlson comorbidity index was associated with physical dependence among stroke patients (Jiménez Caballero et al., 2013). Other studies also suggested that the presence of comorbidities such as heart disease and COPD were risk factors of cognitive impairment among stroke patients (Valkova and Peychinska, 2012).

Specifically, the three comorbidity patterns identified in our study showed generally significant associations with physical and cognitive functional outcomes. These results extended the findings from previous studies of comorbidity patterns and health outcomes among the general populations by disentangling the relationship between comorbidity patterns and functional status among stroke survivors. The heart-gastrointestinal-psychiatric disease and metabolic-kidney disease patterns showed relatively consistent associations with the three measurements of cognitive and physical functional outcomes. Similarly, prior studies indicated the associations between heart diseases and metabolic syndromes with various health outcomes (e.g., morbidity, functional decline, muscular weakness, and cognitive function) among stroke patients (Akbal et al., 2012; Li et al., 2016; Specogna et al., 2017). In contrast, the degenerative-cardiopulmonary pattern was associated with an increased likelihood of moderate-to-severe physical dependence assessed by BI but not by mRS. This may indicate the discrepancy of BI and mRS in monitoring the impact of degenerative-cardiopulmonary diseases on physical function in stroke patients. Prospective follow-up studies will help further clarify the longitudinal associations between comorbidity patterns and post-stroke functional status as well as the concordance between functional outcome parameters in patients with stroke.

Our hospital-based study covered a broad range of chronic conditions in a large sample of stroke patients that were defined by integrating information from face-to-face interviews, clinical examinations, and instrumental and laboratory tests. However, our study also has limitations. First, the cross-sectional nature of the study design does not allow causal inference of the observed associations and mediations, and the findings might be subject to selective survival bias. Thus, caution is needed when interpreting the findings. Second, the study participants from the two local general hospitals might not be representative of the stroke patient population in China, which should be kept in mind when generalizing our findings to a broad patient population. Third, although factor analysis has been most frequently used in the literature to explore comorbidity patterns (Ng et al., 2018), the results may vary with the definition, type, number, and prevalence of included chronic diseases as well as participants’ sociodemographic characteristics. For instance, some common heart diseases (i.e., heart failure, coronary heart disease, and arrhythmia) did not cluster into a single group, as indicated in our study. Therefore, the interpretation of findings should be incorporated with pathophysiological and clinical features of the chronic conditions. Fourth, the prevalence of arthritis might have been underestimated because the misdiagnosis and underdiagnosis of arthritis was fairly common in China, particularly in primary care settings (Tian et al., 2021). Fifth, functional outcomes were assessed within 2 weeks after hospitalization. The findings may be relevant to the functional status of stroke patients in the acute phase and may have implications for early rehabilitation intervention. Future studies that assess cognitive and physical functioning of patients during the period of a few months post-stroke are warranted to confirm the associations between comorbidity and functional outcomes. Finally, we were not able to explore the potential influences of stroke characteristics (e.g., severity, size, and location) on the associations between comorbidities and functional outcomes due to lack of detailed CT or MRI data.

## Conclusion

Our study indicates that comorbidity is highly prevalent among Chinese stroke patients and that chronic conditions are clustered in certain patterns (e.g., patterns of degenerative-cardiopulmonary, heart- gastrointestinal-psychiatric, and metabolic-kidney diseases). Furthermore, the burden and patterns of comorbidity are associated with both poor physical and cognitive function in patients with acute ischemic stroke. These findings may have implications for the proper management of comorbidity among stroke patients in order to maintain and improve post-stroke physical and cognitive functioning outcomes but warrant further investigation in longitudinal studies. Assessments of comorbidity should therefore be routinely included in stroke research and clinical practice.

## Data availability statement

The raw data supporting the conclusions of this article will be made available by the authors, without undue reservation.

## Ethics statement

The studies involving human participants were reviewed and approved by the study protocols from the Ethics Committee at the Jining No. 1 People’s Hospital, Shandong (No. 2015B006). The patients/participants provided their written informed consent to participate in this study.

## Author contributions

RS, ZY, YH, ZZ, YD, JD, BB, and CQ: conception and design of the study. ZY, YH, ZZ, and BB: execution. RS: statistical analysis. RS and CQ: writing the first draft of the manuscript. DV, JL, and CQ: supervision. All authors revised the manuscript.
